# Pyoderma Gangrenosum Precipitated by Breast Engorgement Following Lactation Discontinuation: A Rare Case Report

**DOI:** 10.7759/cureus.42203

**Published:** 2023-07-20

**Authors:** Sarosh Sarwar, Fatima Sajid, Asad Ullah Wasim, Madeeha Subhan Waleed, Pawan Kumar Thada

**Affiliations:** 1 Medicine and Surgery, Fazaia Medical College, Islamabad, PAK; 2 Research, Larkin Community Hospital, Miami, USA; 3 Dermatology, PAF Hospital, Islamabad, PAK; 4 Internal Medicine, Ayub Medical College, Abbottabad, PAK; 5 Medicine and Surgery, Allied Hospital, Faisalabad, PAK

**Keywords:** skin infections, breast ulcers, breast engorgement, necrotic ulcers, isolated pyoderma gangrenosum, infections, cribriform scars, neutrophilic infilteration, pathergy, idiopathic pyoderma gangrenosum

## Abstract

Pyoderma gangrenosum (PG) is an inflammatory disease characterized by recurrent painful ulcers, eventually leading to cribriform scars. PG is mostly a diagnosis of exclusion with neutrophilic skin infiltration. We present a case of a 35-year-old female patient whose first presentation of PG occurred in the first trimester of pregnancy, which recurred after discontinuation of breastfeeding. The patient also had a history of taking prolonged IM and IV analgesics for her chronic migraines. The patient was initially treated with steroids and necessary wound care, during which symptoms remained controlled. However, a year later, the patient presented with an acute flare-up of the disease in her postpartum period, mainly involving her breasts bilaterally. Extensive wound debridement was performed due to the severity of her necrotic ulcers and failure to respond to conservative management, which was followed by partial thickness skin grafting.

## Introduction

Pyoderma gangrenosum (PG) is a rare neutrophilic skin dermatosis characterized by chronic recurring painful necrotic ulcers. These erythematous ulcers are undermined with bluish borders, eventually leading to disfiguring cribriform scars as they progress [1]. PG occurs predominantly in females and usually affects the lower limbs (peritibial areas), hands, feet, and trunk, and has been reported to involve genital mucosa, eyes, spleen, and lungs [2]. It usually occurs in four different variants: classic ulcerative type (most common), vegetative, bullous-pustular, and superficial granulomatous variant [1].

PG is often considered to be idiopathic; however, it has a strong link with inflammatory and autoimmune diseases such as Crohn’s disease, rheumatoid arthritis, inflammatory bowel disease, etc. [3]. Although symptoms of PG often resemble a bacterial infection and therefore were initially considered infectious in origin, recent studies suggest an inflammatory and autoimmune pathophysiology of PG, mostly accompanied by secondary bacterial infections [3].

Recently, pregnancy and the postpartum period have been found to predispose PG, further supporting the inflammatory and immunosuppressive theory of PG origin [4]. Genetic abnormalities have also been found in PG-associated syndromes [3].

Many precipitating factors have been considered to contribute to the development of PG. Some studies have shown that physical trauma is a significant risk factor in PG development [5,6]. Certain drugs like propylthiouracil, isotretinoin, sunitinib, tyrosine kinase inhibitors, TNFα inhibitors, and granulocyte-colony stimulating factor, along with cocaine drug abuse, also play a role in PG development [3,7]. The latest studies show that prolonged external compression of the skin, e.g., seat belt compression, has an important role in precipitating PG [5,8]. Compression of blood vessels causing arterial and venous insufficiency internally has also recently been reported to be a trigger factor in some cases other than external compression of the skin [9,10].

## Case presentation

We present a case of a 35-year-old female patient who presented to the emergency department with pustular to ulcerative lesions affecting her limbs and trunk. The first presentation was during her first trimester of pregnancy in February 2020, with multiple geometric-shaped, painful, ulcerative lesions on the right foot and both hands. According to the patient, all of these ulcers started after minor injuries at home and progressively enlarged to form large necrotic ulcers with purulent discharge. Her husband revealed that the patient had a long previous history of self-administering intravenous and intramuscular analgesics for her chronic migraines. However, in her current flare-up of the disease, no history or evidence was found of any sort of self-induced trauma or parenteral medication administration for past one year.

There was no history of abdominal pain, diarrhea, constipation, weight loss, joint pain, oral ulcers, or photosensitivity. The patient had been treated with Tonoflex (Paracetamol + Tramadol HCl) and Lexotanil (Bromazepam) for the last 15 years for her migraine. The patient was a non-smoker, non-alcoholic, and there was no history of food/drug allergies. She had no history of past surgeries or blood transfusions. The patient was married and had three uncomplicated previous pregnancies.

**Figure 1:**
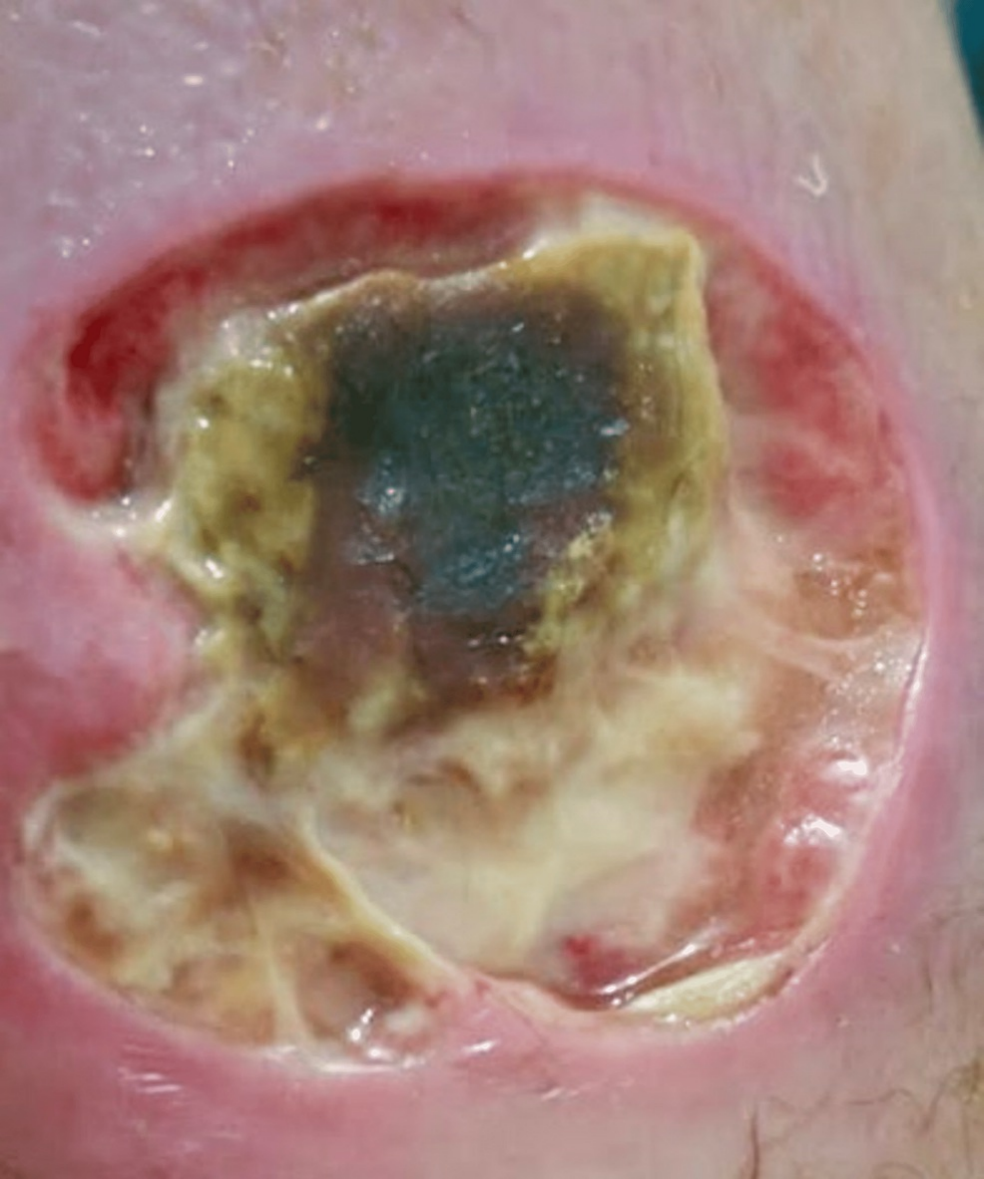
Ulcer (4cm x 5cm) with necrotic purulent base and yellowish discharge on dorsum of the right foot.

**Figure 2:**
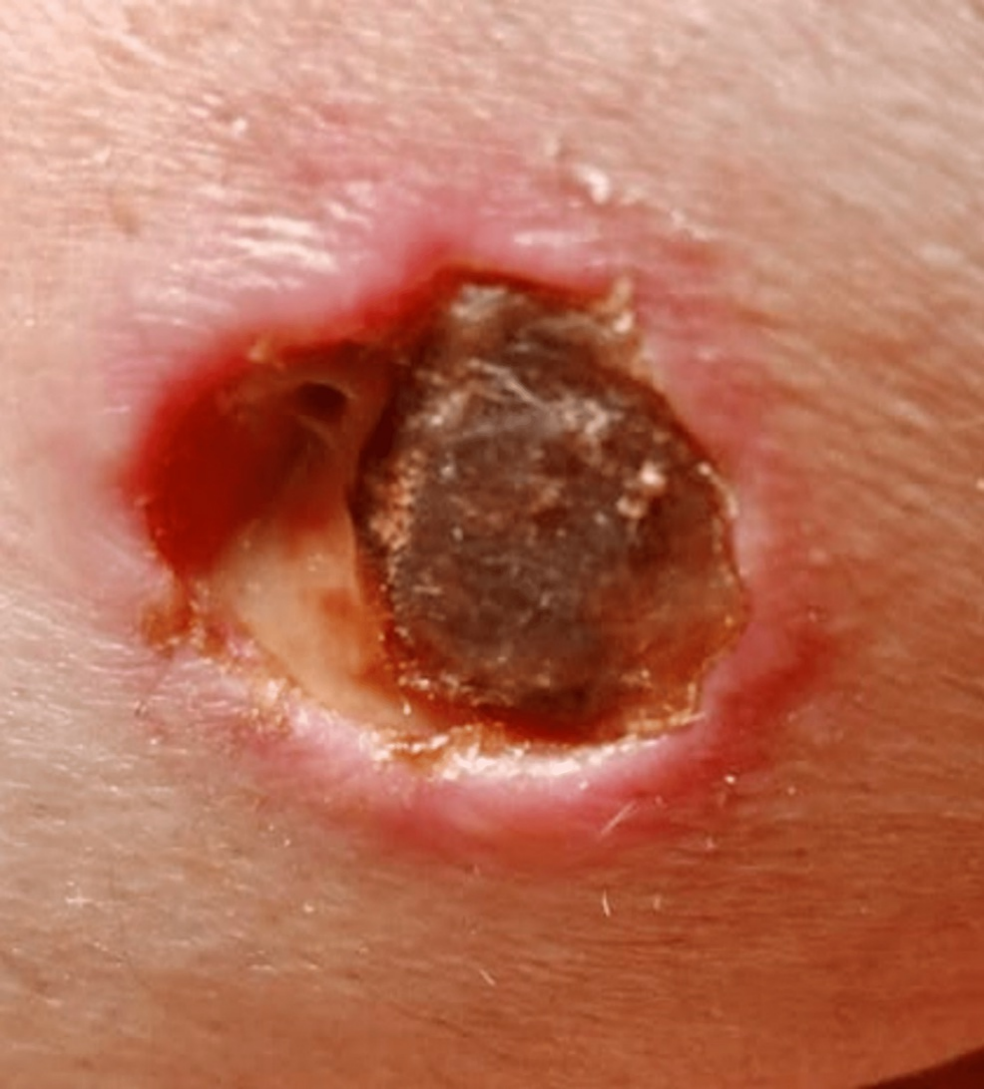
Scarred ulcer (2.5cm x 4cm) with undermined erythematous borders on dorsal aspect of left ring finger.

On examination, the patient had five large necrotic ulcers on the dorsum of her right foot, the volar aspect of her right arm, and the dorsal aspect of her fingers in both hands, with the largest measuring 4 x 5 cm. The ulcers had a necrotic purulent base with yellow discharge, violaceous undermined edges, and a few healed cribriform scars on bilateral lower legs (Figures 1, 2). Anti-Nuclear Antigen antibodies (ANA), extractable nuclear antigens, anti-ds DNA, and workup for anti-phospholipid syndrome were negative. Further workup to rule out Inflammatory Bowel Disease including Blood CP, Serum Albumin, Fecal occult blood, fecal calprotectin and abdominal ultrasound, all came out to be within the normal reference range. Histopathology of the ulcer margin showed an ulcerated epidermis with a thick neutrophilic crust, scattered neutrophilic infiltrate in the dermis, and subcutaneous fat with no evidence of malignancy, suggestive of PG.

 

She was treated with topical potent steroids, oral prednisolone 40 mg per day for two weeks, and analgesics for wound care. The lesions healed after one month with cribriform scarring.

**Figure 3:**
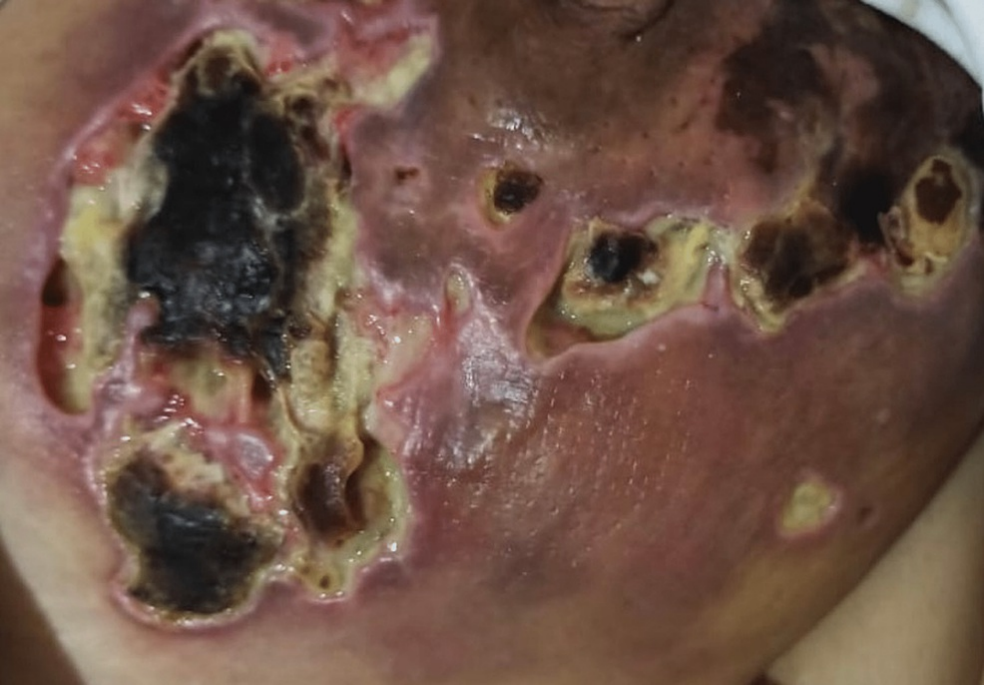
Violacious necrotic ulcers with undermined edges on right breast.

A year later, she presented again with large, painful foul-smelling, necrotic lesions on bilateral breasts that were triggered by breast engorgement after the patient suddenly discontinued breastfeeding her six-month-old child, as shown in Figure 3. Her parenteral administration of analgesics was discontinued since the start of pregnancy. There was no history of physical trauma or any other triggering factor either. On examination, the patient had irregularly shaped deep ulcers circumferentially present on both of her breasts, sparing the nipples and areola, with the widest diameter of 10 cm. A few smaller ulcers were also present in the left inframammary fold. The ulcers had violaceous undermined edges, a necrotic purulent floor, and adherent black necrotic crusts in a few areas. The patient was immediately admitted, and a complete blood picture showed leukocytosis with neutrophilia and elevated CRP. The culture and gram stain of pus swab showed growth of methicillin-resistant Staphylococcus aureus (MRSA) and Escherichia coli (E. coli).

Antibiotic therapy guided by culture sensitivity was started, and based on her history of PG, she was started on 40 mg of oral prednisolone. A calculated decision was taken after a multidisciplinary meeting with the general surgery and plastic surgery department to perform conservative surgical debridement as the patient was not responding well to mechanical debridement alone. The debridement was done to remove all necrotic tissue without disturbing the active margin of the ulcer. The risk of patient's disease getting worsened by surgical debridement due to Pathergy was kept in mind, but considering the severity of her ulcers, the rapid progression, non-responsiveness to other methods of conservative debridement, a careful decision was made to perform surgical debridement while simultaneously starting the patient on oral steroids, followed by Negative-pressure wound therapy with daily vacuum-assisted closure (VAC) dressings.

After waiting two weeks for sufficient granulation tissue to appear, skin grafting was done with partial split skin grafts (PTSG) for which graft was taken from her left thigh. Upper and lower right quadrant grafting were successful however lower left quadrant graft rejection occurred, for which another skin grafting was done and was successful.

## Discussion

PG is a rare neutrophilic dermatosis that is usually a diagnosis of exclusion [7,11]. Although the exact pathogenesis is unknown, many complex pathogenic mechanisms have been described in the development of PG. It can be an idiopathic disease or a part of various systemic conditions, e.g., inflammatory bowel disease and other autoimmune conditions [12,13]. Pathergy is a common finding in PG. Previously, pressure blunt trauma has been described as a factor leading to inflammation and ulceration of the skin in PG development [13].

Pathergy is described as an exaggerated skin response to minor trauma such as needle stick injury, blunt trauma, or pressure [13]. PG triggered by external compression and pressure has been described in very few case reports. Rashid et al. described a case of a middle-aged female who developed PG of the abdomen due to minor compression with the seat belt during a long car ride [8]. Keskin et al. described the development of PG in a battered child that was triggered after minor blunt trauma [5]. To the best of our knowledge, our case is the first description of triggering PG due to internal compression of the skin due to breast engorgement in the absence of any other triggers. We postulate that due to the sudden cessation of breastfeeding, our patient developed breast engorgement leading to increased pressure due to accumulated milk in the breast, which could have produced a similar effect as external compression. This lends credibility to the small pool of evidence that PG can be triggered by both internal and external compression of the skin [5,8,9].

A review of the literature on the existing treatment of PG shows that compression bandage is often the first line of treatment along with immunosuppressants and local wound care [11]. This approach can be problematic in patients whose lesions can be triggered by pressure, so caution must be taken to ensure that the compression bandage is not too tight, as it can induce pathergy in an active PG lesion [11,14]. In a patient with a history of PG, it is pertinent to counsel the patient about the possibility of triggering the lesion due to compression or blunt trauma.

Another important trigger in our patient was pregnancy and the postpartum period. Pregnancy has been known to trigger or exacerbate PG, and the same was found in our patient, where her first presentation with ulcerative lesions was in the first trimester of pregnancy [4]. Many reports of postpartum PG have been reported in the literature, usually complicating a cesarean section incision wound [15]. Cokan et al. described a case of a 23-year-old patient developing PG of the breast eight weeks postpartum, which was preceded by PG in the cesarean section wound. A possible explanation for this phenomenon is the suppression of the immune system during pregnancy [16].

Breast involvement in PG is very rarely reported. Previously reported cases had all presented as complications of surgeries such as reduction mammoplasty, mastopexy, and Port-a-cath insertion [12,17].

Most such cases of PG are misdiagnosed as wound infections, which can lead to a delay in diagnosis and treatment. Our patient's initial presentation of the disease was not severe and included only pain, erythema, and engorgement thus it could initially lead to potential misdiagnosis of mastitis.

Skin grafting has been considered a successful treatment in PG. Morgenstjerne-Schwenck et al. conducted a systematic review in March 2021, showing complete healing in 75.5% of patients with vasculitic ulcers and PG who were treated with skin grafting [18]. Goto et al. wrote a case report on the successful treatment of a 68-year-old woman with PG using oral prednisolone, VAC, and skin grafting combination therapy [11,19]. We followed the same protocol for our patient, which involved initial debridement and antibiotic therapy, followed by the application of VAC dressings for two weeks to allow sufficient granulation tissue to appear. Subsequently, PTSG was performed, using a graft taken from her left thigh. The patient seems to be responding well to the treatment so far, and there are no signs of disease recurrence.

## Conclusions

PG is a painful necrotic skin dermatosis that can severely affect the quality of life. Compressive factors, both external and internal, play a significant role in precipitating the disease. Therefore, caution must be exercised when considering pressure bandages as a treatment, as they can potentially worsen the underlying condition. While the diagnosis of PG is challenging due to the lack of established diagnostic criteria, an early and thorough examination, identification of precipitating factors, and comprehensive laboratory workup can lead to better treatment and overall prognosis.

## References

[1] George C, Deroide F, Rustin M (2019). Pyoderma gangrenosum - a guide to diagnosis and management. Clin Med (Lond).

[2] Teagle A, Hargest R (2014). Management of pyoderma gangrenosum. J R Soc Med.

[3] Gameiro A, Pereira N, Cardoso JC, Gonçalo M (2015). Pyoderma gangrenosum: challenges and solutions. Clin Cosmet Investig Dermatol.

[4] Steele RB, Nugent WH, Braswell SF, Frisch S, Ferrell J, Ortega-Loayza AG (2016). Pyoderma gangrenosum and pregnancy: an example of abnormal inflammation and challenging treatment. Br J Dermatol.

[5] Keskin M, Tosun Z, Ucar C, Savaci N (2006). Pyoderma gangrenosum in a battered child. Ann Plast Surg.

[6] Hadid W, Patel P, Piette WW (2007). After the injury. Am J Med.

[7] Wang JY, French LE, Shear NH, Amiri A, Alavi A (2018). Drug-induced pyoderma gangrenosum: a review. Am J Clin Dermatol.

[8] Rashid RM (2008). Seat belt pyoderma gangrenosum: minor pressure as a causative factor. J Eur Acad Dermatol Venereol.

[9] Freitas VM, Pereira SM, Enokihara MM, Cestari SD (2017). Pyoderma gangrenosum associated with left iliac vein compression syndrome: presentation of difficult diagnosis. An Bras Dermatol.

[10] Wong LL, Borda LJ, Liem T, Keller JJ, Ortega-Loayza AG, Jung E (2023). Atypical pyoderma gangrenosum in the setting of venous and arterial insufficiency. Int J Low Extrem Wounds.

[11] Ashchyan HJ, Nelson CA, Stephen S, James WD, Micheletti RG, Rosenbach M (2018). Neutrophilic dermatoses: pyoderma gangrenosum and other bowel- and arthritis-associated neutrophilic dermatoses. J Am Acad Dermatol.

[12] Asyyed Z, Al-Youha S, LeBlanc M (2018). Pyoderma gangrenosum of the breast after mastopexy. Wounds.

[13] Vernaci GM, Meroni M, Dieci MV (2021). Postsurgical pyoderma gangrenosum in a breast cancer patient: a case report and literature review. Case Rep Oncol.

[14] Fayyaz B (2018). Pyoderma gangrenosum in primary care setting: the challenges involved. J Community Hosp Intern Med Perspect.

[15] Steadman UA, Brennan TR, Daman LA, Curry SL (1998). Pyoderma gangrenosum following cesarean delivery. Obstet Gynecol.

[16] Cokan A, Dovnik A, Žebeljan I, Mujezinović F, Bujas T, Marko PB (2016). Pyoderma gangrenosum in a caesarean wound following caesarean section with late occurrence of pyoderma gangrenosum of the breast. Eur J Obstet Gynecol Reprod Biol.

[17] Inan I, Myers PO, Braun R, Hagen ME, Morel P (2008). Pyoderma gangrenosum after totally implanted central venous access device insertion. World J Surg Oncol.

[18] Morgenstjerne-Schwenck LE, Knudsen JT, Prasad SC (2021). Efficacy and safety of skin grafting in treatment of vasculitic ulcer and pyoderma gangrenosum-a systematic review. Wound Repair Regen.

[19] Goto H, Okada Y, Watanabe S (2021). Successful treatment of ulcerative-type pyoderma gangrenosum with a combination therapy of oral prednisolone, vacuum-assisted closure, and skin grafting. Case Rep Dermatol.

